# Regulatory function of DNA methylation mediated lncRNAs in gastric cancer

**DOI:** 10.1186/s12935-022-02648-1

**Published:** 2022-07-09

**Authors:** Nan Li, Anqi Zeng, Qian Wang, Maohua Chen, Shaomi Zhu, Linjiang Song

**Affiliations:** 1 School of Medical and Life Sciences, Chengdu University of Traditional Chinese Medicine, Chengdu, Sichuan 611137 People’s Republic of China; 2 Institute of Translational Pharmacology and Clinical Application, Sichuan Academy of Chinese Medical Science, Chengdu, Sichuan 610041 People’s Republic of China

**Keywords:** Gastric cancer, DNA methylation, lncRNA, Tumor progression, Gene

## Abstract

As one of the most common malignancies worldwide, gastric cancer contributes to cancer death with a high mortality rate partly responsible for its out-of-control progression as well as limited diagnosis. DNA methylation, one of the epigenetic events, plays an essential role in the carcinogenesis of many cancers, including gastric cancer. Long non-coding RNAs have emerged as the significant factors in the cancer progression functioned as the oncogene genes, the suppressor genes and regulators of signaling pathways over the decade. Intriguingly, increasing reports, recently, have claimed that abnormal DNA methylation regulates the expression of lncRNAs as tumor suppressor genes in gastric cancer and lncRNAs as regulators could exert the critical influence on tumor progression through acting on DNA methylation of other cancer-related genes. In this review, we summarized the DNA methylation-associated lncRNAs in gastric cancer which play a large impact on tumor progression, such as proliferation, invasion, metastasis and so on. Furthermore, the underlying molecular mechanism and signaling pathway might be developed as key points of gastric cancer range from diagnosis to prognosis and treatment in the future.

## Introduction

Gastric cancer (GC) is one of the most frequent malignancies and the third main cause of cancer-related death around the world, which also has shown a poor 5-year survival rate, around 10% for patients with advanced GC over the last decade [1, 2]. Meanwhile, there are various factors contributed to the process of GC, involving multiple genetic and epigenetic alterations [3], which play an essential role in giving rise to the activation of oncogenes or the inactivation of tumor suppressor genes [4]. Otherwise, it is compatible with abundant factors like dietary habits, smoking, and bacterial infection, especially *Helicobacter pylori* (*H. pylori*) infection that was consistent with GC consequently [5, 6]. However, one leading cause to which the outcome might readily relate is the situation that diagnosis early and therapeutic methods are so limited that the effects are not satisfactory. To date, gastroscopy and pathologic diagnosis have been the most reliable strategies as the golden standard of GC in the clinic [7], which needs more advanced approaches considering the high level of death on this issue. Recently, emerging studies about lncRNAs and DNA methylation offer an arena in which more effective diagnosis and treatment guidelines can be developed potentially [8], which is essential for human beings to strive for the favorable duration to overcome it in the future. Therefore, it is time to listen out for more advanced researches beneficial for the diagnosis and treatment of gastric cancer to change the status quo.

**Fig. 1:**
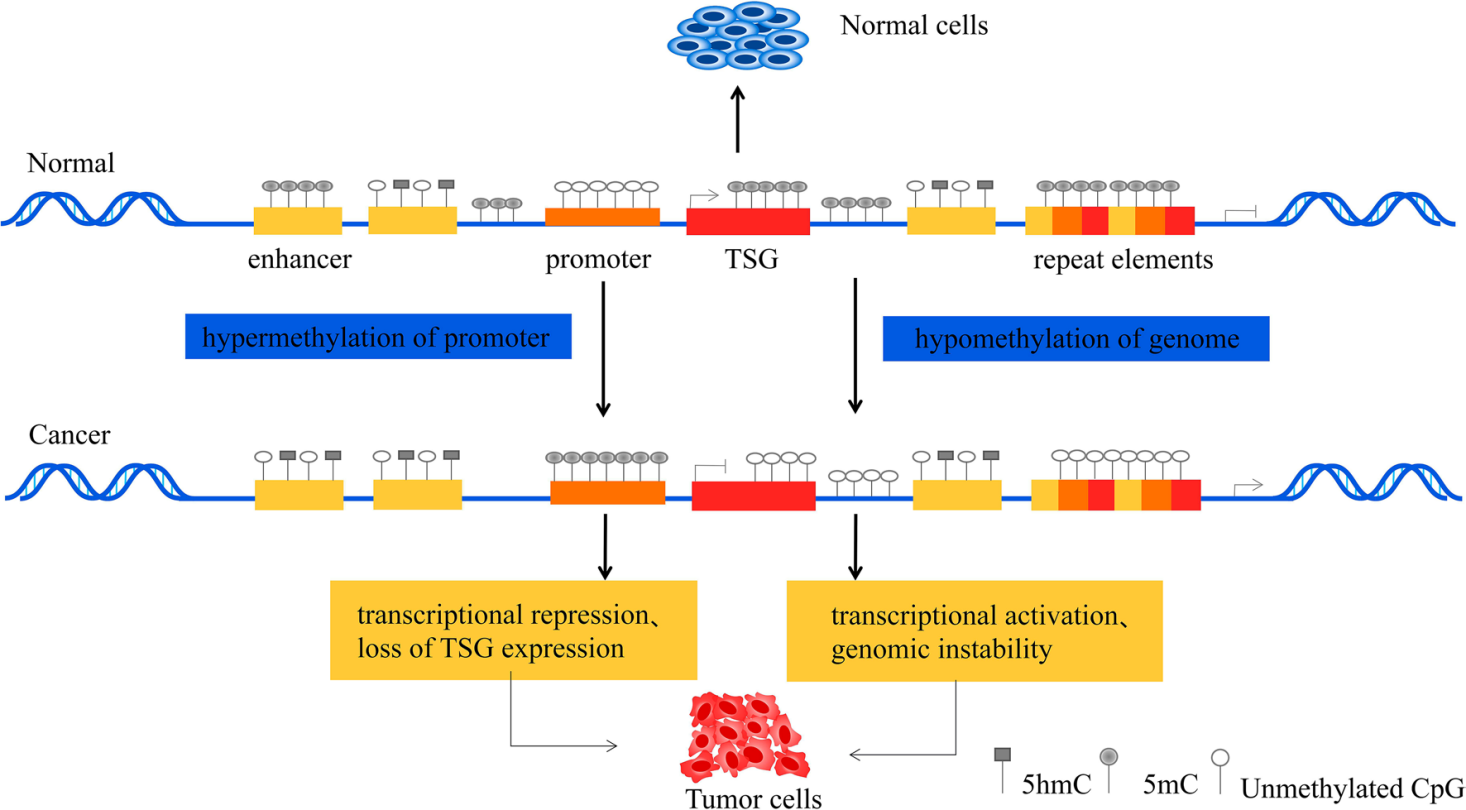
The changes of DNA methylation between young normal cells and aging cancer cells. There is the comparison and discussion between young normal cells and aging cancer cells. In young normal cells, the low methylation and unmethylated CpGs are the preserve of the promoter of tumor suppressor gene (TSG), which spawns the expression of TSG in the wake of producing young normal cells. However, the genome and intergenic regions make it to the higher methylation comparing to the promoter to keep them stable. By contrast, the promoter of TSG was highly methylated in aging cancer cells, obviously resulting in the transcriptional repression as well as loss of TSG expression. What is more, the genome and intergenic regions will be at the opposite stage of hypomethylation in aging cancer cells, which is responsible for the transcriptional activation and genomic instability, oncogenes’ expression holding one example. As a consequence, these abnormal DNA methylations give birth to tumorigenesis finally

DNA methylation, as one of the epigenetic events, which refers to the covalent addition of a methyl group at the 5′ position of the pyrimidine ring of cytosines within the CpG islands, is among the most well-studied and vital epigenetic modification [9, 10]. And there are three enzymes involved in catalyzing DNA methylation process and maintaining genomic methylation patterns, which are DNA methyltransferase 1 (DNMT1), DNMT3A and DNMT3B [11]. It is well-known that DNA methylation is essential for mammalian embryonic development, which means DNA methylation has many functions: it is related to the suppression of transposons and genes, but also to actively transcribe gene bodies, and in some cases, to gene activation itself [12]. However, once aberrant DNA methylation happens, in many cases, can diverse and complex disorders and/or diseases, such as cancer, will creep up on organisms [13]. Although the DNA structure has undergone temporary changes, the genetic information it carries has not changed and will affect gene transcription and expression [14]. Accordingly, the event will prompt the expression or inhibition of proto-oncogenes and tumor suppressor genes (TSG), leaving a crucial mark on the development of cancer, when they suffer abnormal DNA methylation [13]. This means that DNA hypomethylation of oncogenes or DNA hypermethylation of TSG could be served as one kind of barrier to their expression, which, respectively, could trigger tumorigenesis (Fig. 1).

Long non-coding RNA (LncRNA) is known as a length of more than 200 nucleotides(nt) that is capable of encoding the protein rarely, however, a few of them having been demonstrated that some actually could encode small proteins [15–17]. LncRNAs comprise multitudinous classes, such as intergenic transcripts, enhancer RNAs (eRNAs), and sense or antisense transcripts that overlap with other genes [18]. Besides, they also have a variety of functions: relying on their location and their specific interactions with DNA, RNA, and proteins, lncRNA can regulate chromatin function, regulate the assembly and function of membraneless nucleosomes, change the stability and translation of cytoplasmic mRNA, and interfere with signal pathways, many of which will finally affect gene expression in different biological and pathophysiological condition, including cancer [19]. Although, in the first place, many lncRNAs were considered as useless things, they are regarded as the essential substance as more and more researches emerge in gene regulation. Obviously, when it comes to gene regulation, tumor occurrence is an inevitable issue that genome-associated studies have showed a huge number of lncRNAs related to diverse types of cancer [20]. In addition to the abnormal variant in the course of the tumorous initiation and progression, a growing number of researches will be flocked to lncRNAs exceptionally [21, 22]. In a word, lncRNA can not only affect gene expression itself but also regulate gene expression and various signaling pathways by interacting with DNA methylation among a lot of cancers, including gastric cancer [23].

In this review, we collected the literatures on DNA methylation-associated lncRNAs in gastric cancer as well as the probable underlying mechanism between them, which are involved in all processes of gastric cancer. Based on them, we expect an ever-increasing number of exigencies to diagnose and treat gastric cancer to be emerging overwhelmingly.

## Abnormal DNA methylation in cancer

DNA methylation is one of the epigenetic covalent modifications, which appears exclusively from cytosine nucleotides on the CpG islands mostly, and has been described as a vital occurrence in maintaining gene stability and affecting gene expression [24]. Three enzymes are involved in the methylation process and maintenance of methylation patterns, namely DNA methyltransferase 1 (DNMT1), DNMT3A, and DNMT3B [25]. It has been proposed that the ten-eleven translocation (TET) dioxygenases oxidize the 5-methyl group of 5mC to produce 5-hydroxymethylcytosine (5hmC) which is one of the crucial procedures to active DNA demethylation in mammals [26]. Consequently, 5hmC of retrotransposons and satellite repeats and high levels of their transcription are observed in human ageing cancer cells, which play an important in regulating the shutdown of genes expression such as TSGs [27]. Regrettably, its specific mechanism is still unclear. Otherwise, many studies have focused more on the impact of DNA methylation on tumor heterogeneity. For example, it has been demonstrated that DNA methylation can result in the molecular and phenotypic heterogeneity of primary ADCs and lymph node metastases [28]. Obviously, it has been reported that global DNA methylation can reflect spatial heterogeneity [29]. Similarly, the DNA methylation shows extensive heterogeneity in time and space in glioblastoma [30]. Tracing to the source, a unique genome can be found in an ever single cell of the body, which is used to play the role of instructions to produce the components of the organism [24]. In addition, there are two layers of this manual: the DNA sequence itself and methylation modification system related to how to read the information, and it is well-known that every type of cell has the same DNA sequence in an organism, but their methylation patterns have a huge disparity, meaning that DNA methylation is organ and tissue-specific, many genes are hypomethylated in a specific organ but hypermethylated in others [31]. In other words, the methylation patterns might have a significant influence on gene expression. Therefore, how DNA methylation is used in cancer can no doubt be explained with the fundamental interpretation system as mentioned below.

### The activation of oncogenes by DNA hypomethylation

In general, widespread CpGs methylation exists in mammalian genomes, the extent keeping around 70%, which makes genes keep inactive but not turns them off to object strongly to stable genes expression metaphorically like a locking mechanism [31]. Notably, the mammalian genome is mostly composed of methylated repetitive elements, but hypomethylation of them is observed in cancer cells which bring about genetic instability and provoke tumor development, especially expression of oncogenes [32]. Strikingly, DNA hypomethylation to oncogenes has already scattered the malignancy over some cancers, such as breast, ovarian, colorectal cancer, and others. For example, it has been reported that DNA hypomethylation of *Sat2* (juxtacentromeric satellite 2) along with *Sat* (centromeric satellite) is related to early tumor development in breast cancer and tumor progression in ovarian cancer, respectively, also regarding as a potential biomarker of poor progression [31, 33]. Otherwise, DNA hypomethylation of oncogene may occur in different stages of tumorigenesis. For example, DNA hypomethylation of the CDH3 gene promoter, partly due to overexpression of P-cadherin, can induce cell invasion, motility, and migration as well as disillusioning patients’ outcomes in colorectal and breast carcinomas [34–36]. In addition, melanoma associated antigens genes(MAGE), one kind of oncogenes, has become prevalent mostly because of DNA hypomethylation, based on previous studies that showed the impact of MAGE has become more visible on melanoma, lung, breast, bladder, gastric cancer and so on [37].

### The inactivation of tumor suppressor genes by DNA hypermethylation

Normally, TSG promoters are at hypomethylation status to express commonly, but it is observed that they will have a higher level of DNA methylation when it comes to tumorigenesis, which is a characteristic of several cancers and prompts decreased expression of tumor suppression genes [38]. What is more, the hypermethylation of promoters is always mediated by de novo methylases: DNMT3A and 3B are responsible for variation in focused sites, comparing with normal cells that are labeled with a protein complex called polycomb that serves as a repressor by leading to local heterochromatinization [39]. According to more and more studies, tremendous genes were reported to undergo hypermethylation in various cancers. As a consequence, these famous genes were involved in cell cycle regulation, DNA repair, apoptosis, drug resistance, detoxification, differentiation, angiogenesis, and metastasis [13]. For example, the promoter hypermethylation of the adenomatous polyposis coli (*APC*) gene, a tumor suppressor gene, could result in abnormal cell proliferation, cell migration, cell adhesion, cytoskeleton reorganization, and chromosome stability in breast and lung carcinomas [24]. Estrogen receptor (ER) plays an important role in breast cancer, and endocrine therapy is one of the key points during the treatment of breast cancer. However, it is obvious that endocrine therapy suffers from hormone resistance while hypermethylation of ER gene is found [40, 41]. At the same time, gene *BRCA1* in breast and ovarian cancer was uncovered that the level of hypermethylation was clear, which was responsible for DNA repair, transcription, and recombination [42].

Taken together, abnormal DNA methylation in cancer has been a topic of intensive research that the hypomethylation of the oncogenes and the hypermethylation of TSG promoters are enchanted in this field. On one hand, hypomethylation induces oncogenesis by activating oncogenes as well as causing interruption of expression of nearby genes. On the other hand, promoter hypermethylation, in most cases, makes it difficult for tumor suppressor genes to express, resulting in tumorigenesis. Totally, these two types of abnormal DNA methylation occupy a significant position in cancer.

## The roles of lncrnas in cancer

**Fig. 2:**
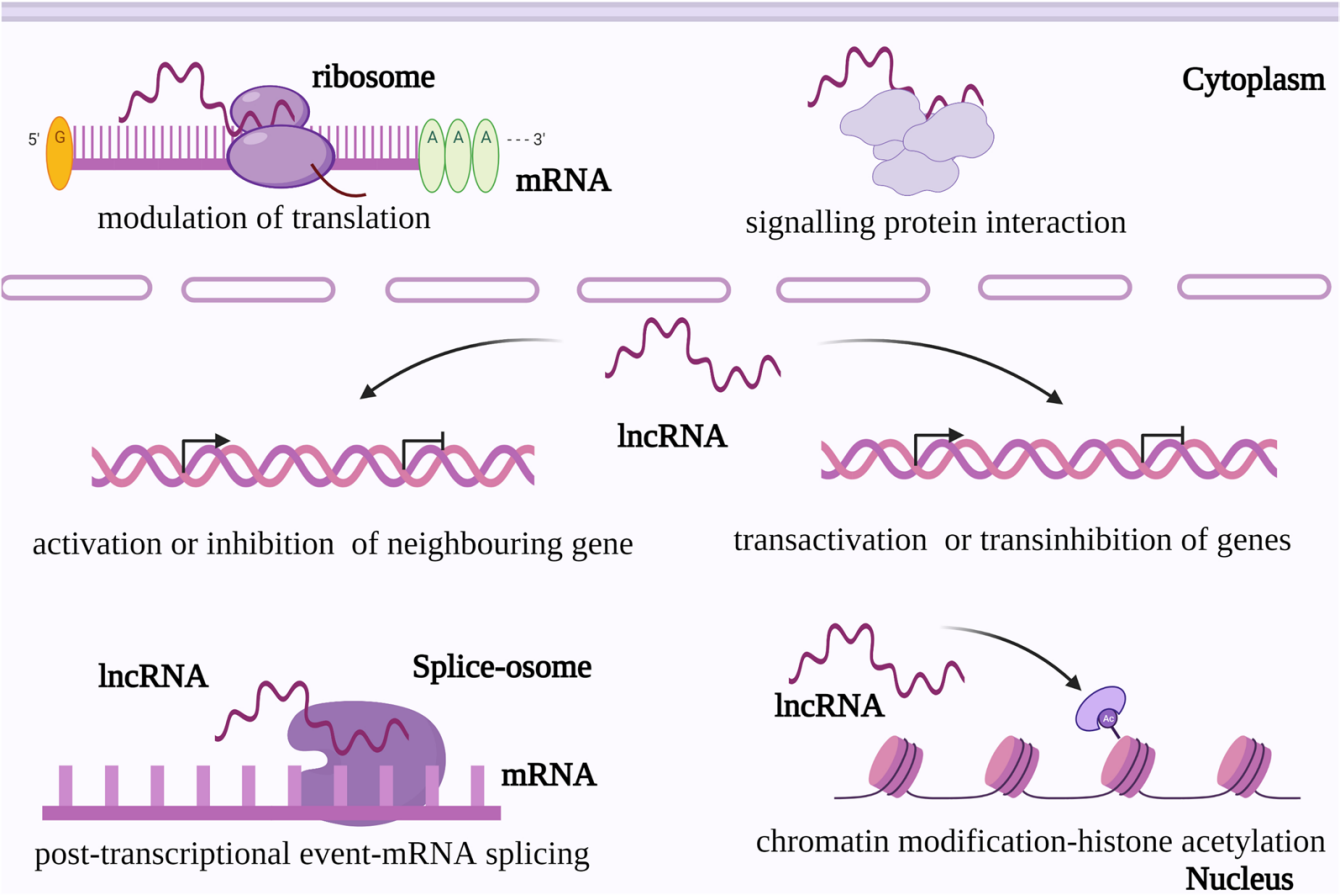
The roles of lncRNAs in cancer. There are main layers of the roles of lncRNAs in cancer. First of all, lncRNAs are able to activate or inhibit their neighboring genes, similarly playing on transactivation or transinhibition of other genes. Once the condition happened, can the genes expression swarm with fatal errors not least because of disordered expression. Secondly, it is inescapable that lncRNAs are bound to exert an influence on the post-transcriptional event, like mRNA splicing, which is regarded as a kind of regulator to interact with splice-osome. Otherwise, their chromatin modification roles also deserve attention, for example, H3K27 trimethylation is closely related to the abnormal proliferation of cancer cells. Moreover, lncRNAs are not confined to the nucleus, for their roles even extend to the cytoplasm, from modulation of translation that can act on ribosomes to signaling protein interaction that has become a vital part of cancer. Overall, lncRNAs are already having broad effects well beyond this RNA itself. And the roles of lncRNAs are being lightened as their researches are uncovered gradually

Thousands of Long non-coding RNAs with a length of more than 200nt that cannot be translated into proteins during the transcription of the genome, then, exhibiting wide distributions in the nucleus and cytoplasm [43]. Although the number of functional lncRNAs is controversial, there are mounting pieces of evidence that lncRNAs have distinct biological functions, such as neurological disorders, immune dysfunction, and cancer [19]. Simply, there are main layers for the basal mechanism of lncRNAs in cancer: some lncRNAs can affect the transcription of neighboring genes, as well as the structure and function of chromatin, and the other function out of their location as regulatory or structural elements to involve in transcription, translation, and signal pathways and so on [19] (Fig. 2). Here, we highlight the oncogenic and tumor-suppressive functions of lncRNAs.

### The oncogenic role of lncRNAs

The oncogenic role, significantly, affecting the occurrence and development of various tumor cells, it has been revealed that lncRNAs interact with different mechanisms to mediate oncogenic expression. For example, lncRNA LINC01139 is one of the oncogenes responsible for immune escape by causing abnormal expression of proteins in the antigen presentation system, which participates in the degradation of the antigen peptide-loading complex and the tumor suppressors RB and p53 [44]. Another classic example is oncogenic lncRNA PCAT19 that promotes the development of tumor cells in prostate cancer, which cooperates with MYC, a transcription factor, to induce the expression of MYC and reduces the probability of damaged DNA strands to be repaired by oppressing expression of the breast cancer 2 gene [45, 46]. Intriguingly, the lncRNA gene *PVT1*, an oncogene overexpressed in cancers, is associated with MYC alike. And it has been shown that the alternative transcript PVT1B, during genotoxic stress, is induced by p53 and contributes to p53-mediated tumor growth restriction in vivo through controlling the adjacent MYC genes’ transcription [47]. Otherwise, the lncRNA LINC02525 has been also demonstrated that its oncogenic role keeps obvious, which is upregulated in neuroblastomas and interacts with the ribosomal protein RPL35 to activate the translation of E2F1, consequently, maintaining the stability of NMYC protein and inducing ERK protein phosphorylation [48].

### The suppressive role of lncRNAs

Ever-increasing shreds of evidence have demonstrated that lncRNAs remain an intimate connection with cancers playing suppressive roles. For example, lncRNA DIRC3 has searched out that its expression was at a lower level correlated with patients’ reduced survival rate in melanoma, the further study showing that it can activate transcription of tumor suppressor IGFBP5 through changing local chromatin structure [49]. In addition, p53, a transcription factor famous as the guardian of the cell's homeostasis, starts a tumor suppressor system that includes a lot of lncRNAs, some of which p53-regulated lncRNAs are downregulated in colorectal cancer [50, 51], suggesting their tumor suppressive roles. Meanwhile, the lncRNA LINC00261, which has been displayed that its expression is extremely lower than normal tissue samples in various cancers, including liver, breast, and gastric cancer, takes part in the DNA damage response in lung cancer, and it can slow down proliferation by triggering G2–M cell cycle arrest [52]. Furthermore, the suppressive effect of lncRNA can also be expressed in regulating signally. For instance, the decreased expression of lncRNA DRAIC in castration-resistant advanced prostate cancer can inhibit its progression by inhibiting the activation of nuclear factor-κB (NF-κB) by intervening in inhibitors of NF-κB kinase (IKK) activity. It restricts interaction between IKK and the subunits of the IKK complex, thereby failing to activate NF-κB [53].

### LncRNAs’role in tumor growth, microenvironment drug, resistance

Except what we mentioned above, there are some interaction between lnRNAs and tumor growth as well as microenvironment. For instance, it has been verified that LncRNA CERS6-AS1 can active the ERK signaling pathway resulting in proliferation and metastasis in pancreatic cancer [54]. Furthermore, lncRNA RAB11B-AS1 caused by hypoxia has an important impact on tumor angiogenesis and metastasis by increasing the expression of angiogenic factors in hypoxic breast cancer [55]. LncRNA AC020978 can be upregulated under the conditions of glucose starvation and hypoxia in non-small cell lung cancer, which could improve the PKM2 (Pyruvate kinase isozymes M2) protein stability [56]. It has been revealed that lncRNA BX111 transcription was induced by hypoxia-inducible factor (HIF-1α), and then BX111 can contribute to proliferation and invasion of pancreatic [57]. LncRNAs have close link with drug resistance in cancer. For example, it has been reported that the expression of lncRNA H19 was considerably upregulated in tamoxifen-resistant breast cancer cell line and tumor tissues, and knockdown of H19 enhanced the sensitivity to tamoxifen both in vitro and in vivo [58].

In Sections 1, 2, 3, we, firstly, introduced that GC is frequent malignancies and the third main cause of cancer-related death around the world [1]. After that we realized that there are some close connections between DNA methylation as well as lncRNAs and GC. Consequently, as we mentioned above, cancers are associated with abnormal methylation, which involves in hypomethylation and hypermethylation. Meanwhile, the roles of lncRNAs need to be highlighted in cancers too, including oncogenic and suppressive roles.

## Crosstalk between lncRNA and DNA methylation in cancer

The mutual interaction between DNA methylation and lncRNA has been found to be involved in cancer. Firstly, lncRNAs play an important role in regulating DNA methylation. Usually, lncRNAs act as regulators to affect the methylation of related genes, thereby affecting the signaling pathways responsible for tumorigenesis, for example, it has been indicated that lncRNA MAGI2-AS3 may act as a vital element in inhibiting the DNA methylation of the MAGI2 promoter region, thus upregulating the expression level of MAGI2 and blocking the AKT pathway as well as the Wnt signaling pathway in breast cancer [59]. Otherwise, DNA methylation could regulate the expression of lncRNAs also. The DNA methylation of lncRNAs genes can upregulate or downregulate the expression level of lncRNAs, and then getting in touch with cancer, for instance, the hypomethylation of lncRNA SOX21-AS1 promoter region was associated with the severity in cervical cancer [60].

**Fig. 3:**
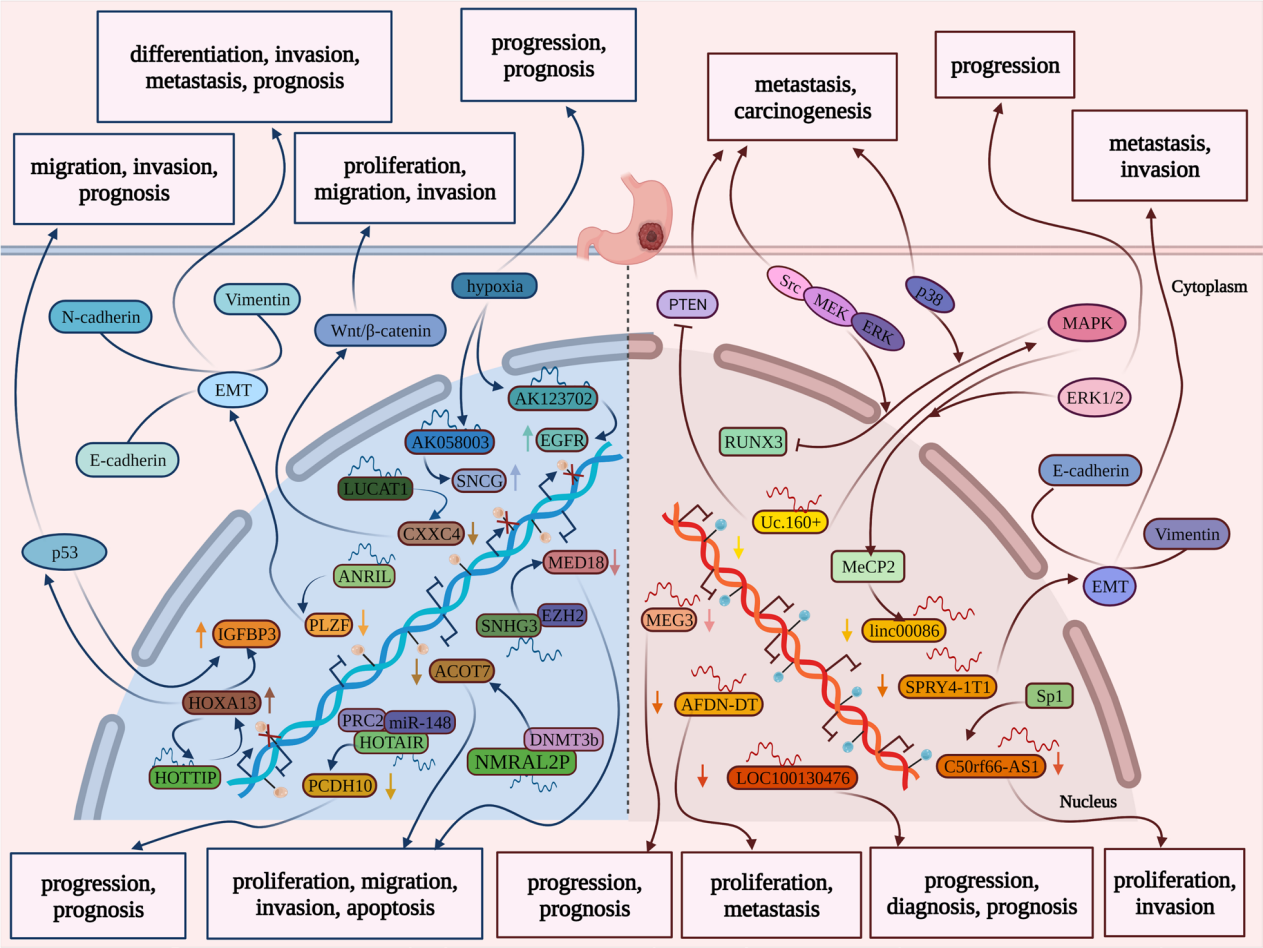
The mutual roles of DNA methylation and lncRNAs for tumor progression in gastric cancer. As shown on the right, abnormal DNA methylation regulates the expression of lncRNAs as tumor suppressor genes in gastric cancer. LncRNA Uc.160+ and lncRNA linc00086 are hypermethylated in GC cells via, respectively, evoking *Src*/MEK/ERK and p38 pathway and regulating MeCP2 by ERK1/2 signaling pathways. Uc.160+ could contribute to the repression of PTEN expression, and MAPK signaling pathway could bring about the inactivation of RUNX3 via evoking Src/MEK/ERK and p38 pathway. SPRY4-IT1 is hypermethylated in GC cells resulting in tumor progression due to the EMT attributed to decrease of E-cadherin and increase of Vimentin. C5orf66-AS1 is hypermethylated in GCA tissues and cell lines, which is associated with its lower expression possibly through abrogating Sp1 binding. LncRNA MEG3, lncRNA AFDN-DT, and LOC100130476 are hypermethylated in GC. Their endings are shown in the figure. On the other side, lncRNAs regulate the abnormal DNA methylation of tumor-related genes in gastric cancer. The PLZF is hypermethylated through regulation of LncRNA ANRIL remarkably in GC tissues and cell lines, and the progression derives from EMT that is negatively correlated with E-cadherin but positively with Vimentin and N-cadherin. MED18 is hypermethylated through regulation of lncRNA SNHG3 by binding to EZH2. PCDH10 is hypermethylated by lncRNA HOTAIR through interacting with miR-148 and DNMT1 in GC. ACOT7 was hypermethylated by lncRNA NMRAL2P through binding to DNMT3b in GC cells. LncRNA LUCAT1 induces hypermethylation of CXXC4 which can regulate Wnt/β-catenin signaling closely related to the development and progression of GC. LncRNA HOTTIP enabled HoxA13 to be hypermethylated and it can be the target of HoxA13 reversely. And IGFBP-3 could interact with HoxA13 as its downstream target in GC and can be modulated by p53 pathway. LncRNA AK058003 is upregulated by hypoxia and regulate the hypomethylation of SNCG. Similar to AK058003, lncRNA AK123702 is also upregulated by hypoxia and capable of regulating the hypomethylation of EGFR in GC cells. Their endings are shown in the figure

As we mentioned previously, it has been demonstrated that a wide range of cancers arises from abnormal DNA methylation [13]. Similarly, accumulating pieces of evidence indicate that DNA hypermethylation of the tumor suppressor gene and DNA hypomethylation of oncogenes tend to induce multistep carcinogenesis in GC [61]. Meanwhile, a huge amount of studies has addressed that lncRNAs could be the emerging factors as tumor suppressor genes and/or the imperative regulators to DNA methylation in various cancers including GC [62]. Tumor development involves multiple processes, such as proliferation, metastasis, invasion, and other complex processes as well as the survival of patients [63], which, partly, has a bearing with abnormal DNA methylation that induces downregulation of lncRNAs as tumor suppressor gene and/or can be affected by lncRNAs as regulators in GC. Hence, abnormal DNA methylation that not only inhibits the expression of lncRNAs as tumor suppressor genes but its methylation processes can be regulated by lncRNAs in gastric cancer will be headlined here, which have been summarized in Fig. 3.

### Abnormal DNA methylation regulates the expression of lncRNAs as tumor suppressor gene in gastric cancer

Many lncRNAs take a significant part in different biological aspects in GC. Various lncRNAs which were functioned as tumor suppressor gene were influenced by abnormal DNA methylation in gastric cancer (Table 1). For example, Uc.160, one of the ultraconserved regions (UCRs), produced transcribed ultraconserved regions (T-UCRs), a novel class of long noncoding RNAs, has CpG islands on its encoding region’s upstream in which the hypermethylation is cancer-specific [64, 65]. It was reported that Uc160+ —If a T-UCRs produced by UCR corresponds to the sense genomic sequence will be named “ + ” and the other corresponds to the complementary sequence will be named “ + A”—was suppressed through the hypermethylation of CpG islands upstream of Uc.160+ compared with the normal stomach samples in gastric cancer, which is related to the growth of tumor cells, finally leading to tumor proliferation [62]. Subsequently, further discussion was performed to find out the regulatory mechanisms, suggesting that Uc.160+ could participate in the activation of the MAPK signaling pathway plausibly, which brings about the inactivation of runt-related transcription factor 3 (RUNX3) via evoking Src/MEK/ERK and p38 pathway and contribute to the repression of PTEN expression [66]. Furthermore, another research revealed that small integral membrane protein 10 like 2A (linc00086) expression was lower in GC tissues compared with normal tissues regulated by DNA methylation resulted from regulation of MeCP2(Methyl-CpG binding protein 2) via extracellular signal-regulated kinase 1/2 signaling pathways (ERK1/2), which, alike, is involved in cancer progression through binding methylated CpG islands [67]. And silencing MeCP2 may induce the expression of linc00086, which may suggest that the downregulated expression of linc00086 in GC is associated with DNA methylation. Meanwhile, another LncRNA, called AFDN-DT(AFDN divergent transcript), also acts as a tumor suppressor in GC which showed a low level in patients with GC [68]. Further study revealed that it was the hypermethylation of the AFDN-DT promoter that was considered as one of the basic mechanisms caused the downregulation of AFDN-DT rendering the proliferation and metastasis of GC *in* xenograft *vivo* [69]. Been firstly reported in melanoma cells as a tumor suppressor, SPRY4-IT1 (SPRY4 intronic transcript 1), a long-coding RNA stemmed from an intron within SPRY4 gene, has been proven that exists in many other cancer cells, like kidney cancer and esophageal cancer [70–72]. Intriguingly, it was proposed that SPRY4-IT1 was extremely reduced in GC cells resulting in proliferation, invasion, and metastasis in vitro and *in* xenograft *vivo* due to the Epithelial-mesenchymal transition attributed to a decrease of E-cadherin and increase of Vimentin mostly and SPRY4-IT1 promoter region was highly hypermethylated, which restricts its expression [73].

**Table 1:** The influences of DNA methylation in LncRNAs’ in gastric cancer

LncRNA	Pattern	Expression level	Mechanism	Outcomes
Uc.160+	Hypermethylation	↓	Src/MEK/ERK and p38 pathway	Metastasis, carcinogenesis
Linc00086	Hypermethylation	↓	ERK1/2 signaling pathway	Progression
SPRY4-1T1	Hypermethylation	↓	EMT	Metastasis, invasion
C50rf66-AS1	Hypermethylation	↓	Abrogating Sp1 binding	Proliferation, invasion
MEG3	Hypermethylation	↓	–	Progression, prognosis
AFDN-DT	Hypermethylation	↓	–	Proliferation, metastasis
LOC100130476	Hypermethylation	↓	–	Progression, diagnosis, prognosis

Gastric cardia adenocarcinoma (GCA) evidently, is featured in the aberrant process. For example, as an example of the tumor suppressor gene, another lncRNA MEG3, located at 14q32, was significantly downregulated in GCA patients and cell lines, and overexpression inhibited GCA cell proliferation and invasion in vitro. In addition, the proximal promoter and enhancer region hypermethylation and dysregulation of MEG3 were associated with poorer GCA patients’ survival [74]. Similar to MEG3, C5orf66-AS1, a long non-coding RNA as a tumor suppressor gene, is located at 5q31.1. It was investigated that C5orf66-AS1 was significantly downregulated in GCA tissues and cell lines. And further methylation analysis demonstrated that the aberrant hypermethylation of the regions around the transcription start site of C5orf66-AS1 was more tumor specific and was associated with its lower expression possibly through abrogating Sp1 binding, which expression level was related to tumor progression, including poorer GCA patients’ survival [75]. Different from the above, although lncRNA LOC100130476 serves an important function on the progression of GCA through its hypermethylation at CpG island critical for gene silencing, involving TNM stage, differentiation, and metastasis, the underlying mechanism has not been explained [76]. The findings suggested that aberrant DNA methylation of lncRNA C5orf66-AS1, MEG3, and LOC100130476 may play important roles in GCA tumorigenesis and C5orf66-AS1 and MEG3 may serve as a potential diagnostic and prognostic marker in predicting GCA patients’ survival.

### LncRNAs regulate the abnormal DNA methylation of tumor-related genes in gastric cancer

An increasing number of lncRNAs have been identified as regulators that play a significant role in the regulation of DNA methylation in GC (Table 2). For example, promyelocytic leukemia zinc finger (PLZF), primarily confirmed as a gene fused to RARa in acute promyelocytic leukemia patients and functioned as a tumor suppressor, has been demonstrated that it wasn’t only involved in hematological malignancies but in various solid tumors, such as hepatocellular carcinoma, pancreatic and lung cancer, thyroid carcinoma, prostate, and gallbladder cancer, encompassing the multistep of cancer development including proliferation, invasiveness, motility, and resistance to apoptosis [77–87]. ANRIL (CDKN2B antisense RNA 1), a long non-coding RNA associated with coronary disease, intracranial aneurysm, type 2 diabetes, and cancers, is transcribed in the opposite direction from the INK4b-ARF-INK4a gene cluster [88, 89]. As previously mentioned and unexplored value in gastric cancer, it was identified in xenografts experiment and in vivo study that the expression of PLZF was remarkably low in GC tissues and cell lines responsible for GC cell proliferation and migration derived from EMT that is negatively correlated with E-cadherin but positively with Vimentin and N-cadherin, negatively related to ANRIL which indirectly caused DNA methylation of PLZF promoter through recruiting PRC2 (polycomb repressive complex 2), especially EZH2(histone-lysine N-methyltransferase enzyme) which is the functional enzymatic component of the PRC2 both in vitro and in vivo [90]. And ANRIL knockdown can block the effects of TET2 on gastric cancer cell proliferation and colony formation [91]. Otherwise, small Nucleolar RNA Host Gene 3 (SNHG3), a novel Long non-coding RNA, has been validated that it was concomitant with a variety of cancers, aimed at malignant progression as well as prognosis in vitro and *in* xenograft *vivo*, such as hepatocellular carcinoma [92], colorectal cancer [93], ovarian cancer [94]. Accordingly, it was proposed that SNHG3 also had a significant impact on the positive progression of GC in the study that displayed that MED18, a tumor suppressor in respect to proliferation, migration, and invasion of GC, was downregulated by SNHG3 epigenetically via methylating MED18 promoter by binding to EZH2 [95]. Except for the PRC2 family, DNA methyltransferase and mi-RNAs also make a pivotal difference. For instance, long non-coding RNA HOX transcript antisense intergenic RNA (HOTAIR) served as a regulatory factor, exerted epigenetic effects on progression in diverse cancers as well as GC [96]. It was found that HOTAIR had an influence on PCDH10 renowned as a tumor suppressor gene, which suffered a methylated alteration resulted by HOTAIR through interacting with miR-148 and DNMT1, bringing about oncogenic changes in GC [96]. Moreover, Acyl-CoA thioesterases (ACOTs) play an important role in the degradation of fatty acyl-CoA which participates in plentiful metabolic processes in humans [97, 98]. ACOT7, one of the members of the ACOTs family, is necessary to cell cycle progression in pulmonary carcinoma [99]. Hence, it was confirmed that lncRNA NMRAL2P showed overexpression in GC cells, inhibiting proliferation, migration, and invasion which was linked to DNA methylation of ACOT7 promoter, NMRAL2P regulating by binding to DNMT3b [100]. Long non-coding RNA LUCAT1, known as lung cancer associated transcript 1, has been demonstrated to be engaged in the development of many cancers, such as clear cell renal cell carcinoma, non-small lung cancer, glioma, osteosarcoma, and colorectal cancer. Similarly, it was investigated the functions and molecular mechanisms of LUCAT1 in the carcinogenesis of GC. It was found that LUCAT1 induces methylation of CXXC4 (CXXC finger protein 4), a negative regulator of Wnt/β-catenin signaling and SFRP2, thereby regulating Wnt/β-catenin signaling whose alteration is closely related to the development and progression of GC [21]. HOTTIP lncRNA, a component of the HomeoboxA cluster in its 5'-end, manipulated multiple processes of cancer like facilitating the proliferation of pancreatic cancer cells [101], and metastasis of hepatoma [102]. Interestingly, it was verified that HOTTIP enabled HoxA13 from Homeobox genes family concerned with migration and invasion of GC to express actively relying on DNA hypomethylation of HoxA13 promoter partly and it can be the target of HoxA13 reversely *in* xenograft *vivo* [103]. Furthermore, in-depth research revealed that the insulin-like growth factor-binding protein-3(IGFBP-3), famous as a fundamental regulator in many signaling pathways, interacted with HoxA13 as its downstream target in GC and can be modulated by the p53 pathway. Therefore, the HoxA13–HOTTIP–IGFBP-3 axis may be considered as the underlying mechanism in GC [103].

**Table 2:** Crosstalk between LncRNAs and DNA methylation of cancer-related genes in gastric cancer

LncRNA	Gene	Pattern	Expression level	Mechanism	Outcomes
HOTTIP	HOXA13	Hypermethylation	High level	HOXA13-HOTTIP-IGFBP3 axis/p53	Migration, invasion, prognosis
AK058003	SNCG	Hypermethylation	High level	Hypoxia-AK058003-SNCG axis	Migration, invasion
AK123702	EGFR	Hypermethylation	↑	Hypoxia-AK123702-EGFR axis	Migration, invasion
ANRIL	PLZF	Hypermethylation	↓	EMT	Differentiation, invasion, metastasis, prognosis
LUCAT1	CXXC4	Hypermethylation	↓	Wnt/β-catenin	Proliferation, migration, invasion
SNHG3	MED18	Hypermethylation	↓	SNHG3/EZH2-MED18 signaling	Proliferation, migration, invasion, apoptosis
NMRAL2P	ACOT7	Hypermethylation	↓	-	Proliferation, migration, invasion, apoptosis
HOTAIR	PCDH10	Hypermethylation	↓	-	Progression, prognosis

Oxygen is essential for the energy metabolism that drives cell biology, lower oxygen levels leading to more aggressive tumor cells. As a consequence, suffering hypoxia is not just considered to be a key microenvironmental factor that induces cancer metastasis but related to changes in gene expression [104]. There is no doubt that the proceeding also presides over tumor metastasis in GC. For example, it was found that lncRNA AK058003 is upregulated by hypoxia, which is responsible for GC migration and invasion in vivo and in vitro. Otherwise, it was demonstrated that AK058003 is able to regulate the expression of metastasis-related gene γ-synuclein (SNCG), the further study showing that SNGG gene CpG island was hypomethylated while AK058003 was deleted. Furthermore, SNCG expression is also related to hypoxia inducing hypoxia-induced GC cell metastasis. Consequently, the hypoxia/lncRNA-AK058003/SNCG pathway may be a novel signaling pathway in GC [105]. Surprisingly, another study found that the role of lncRNA AK123702 was similar to AK058003, suggesting that AK123702 was upregulated by hypoxia resulting in metastasis and invasion in vivo and *vitro*, and metastasis of hypoxia-induced was mediated by AK123702 in GC cells alike. In addition, AK123702 regulated positively the expression of metastasis-related gene EGFR whose expression was also increased by hypoxia, and upregulation of EGFR by AK123072 could mediate hypoxia-induced metastasis in GC cells. Significantly, the EGFR gene CpG island was also hypomethylated in GC cells expurgated AK123702. As a consequence, the hypoxia/lncRNA-AK123072/EGFR pathway maybe another novel signaling pathway in GC [106].

## Conclusion and perspective

Gastric cancer places a dominant position in the majority of cancers in human’s life unfolding that people in the world are frequently confronted with statements about the alarmingly high death rate of GC, most of them having been diagnosed at a late stage, which results from a sharp spell of limitation including diagnostic and therapeutic methods as well as poor outcomes [2, 7]. Therefore, advances of new biomarkers of diagnosis and treatment specific to GC deserve to be taken more seriously. Recently, attention has multiplied about DNA methylation and/or lncRNAs as the emerging and promising approaches [107], which provides us with priceless insights to elicit deeply the more effective and non-invasive ways to alter the current dilemma of GC, showing that their advantages could contribute a huge opportunity to the diagnostic and therapeutic strategies. Unfortunately, there are some existing circulating biomarkers for GC diagnosis and prognosis, but they always show low sensitivity and specificity, furthermore, gastrointestinal endoscopy being one of the most effective procedures for GC diagnosis [1]. Consequently, a growing number of studies are focused on finding more valid biomarkers released from the tumor tissues into body fluids such as blood, urine, stomach juice and so on to alter these drawbacks [108]. Fortunately, some circulating molecules such as lncRNAs have been found that they can be used to developing new measures for early diagnosis of GC with a sensitivity close to 80% [1, 109]. Not only that, it has been verified that DNA methylation as one of the epigenetic modifications can be another strategy for cancer therapy [110]. However, due to arising from a sequence of genetic and epigenetic molecular events, the strategy needs the combination of multiple genetic and epigenetic targets.

The silver lining may have a cloud attached. It is obviously struggling to apply them to the clinic as the biomarkers, and overcome difficulties about intratumoral heterogeneity inherent in human cancers [24]. Meanwhile, so intricate and indistinct is the underlying mechanism that more and more difficulties need to be thrown off. Although the spiking number of challenges, we have already accumulated a lot of fundamental knowledge and research about DNA methylation and/or lncRNAs in GC. Consequently, there are more expectations for the future of the improvement and development of novel biomarkers of DNA methylation and/or lncRNAs in GC. Sustained collaborative efforts may soon lead to significant progress in changing the evolution of this disease.

## Data Availability

Not applicable.
